# In Vitro Evaluation of Ruminal Fermentation and Methane Production in Response to the Addition of Modified Nano-Bentonite with or Without *Saccharomyces cerevisiae* to a Forage-Based Diet

**DOI:** 10.3390/ani15142081

**Published:** 2025-07-15

**Authors:** Sohila Abo-Sherif, Sobhy Sallam, Ali M. Allam, Mounir El-Adawy, Yosra Soltan

**Affiliations:** Department of Animal and Fish Production, Faculty of Agriculture, University of Alexandria, Aflaton St., El-Shatby, P.O. Box 21545, Alexandria 21526, Egypt; sohilas629@gmail.com (S.A.-S.); soubhy.salam@alexu.edu.eg (S.S.); aallam04@yahoo.com (A.M.A.); dr.eladawy@yahoo.com (M.E.-A.)

**Keywords:** yeast, nano-modified clays, fiber degradability, methane

## Abstract

Modified nano-clays, alone or combined with probiotics, may offer a novel and sustainable approach to improve ruminal fermentation and mitigate CH_4_ emissions in high-fiber diets. This study evaluated the properties and effects of modified nano-bentonite (MNB), with or without yeast (*Saccharomyces cerevisiae*), compared to natural bentonite (NB) and monensin, using the in vitro gas production (GP) technique. The substrate used was a basal diet composed primarily of forage (*Trifolium alexandrinum* clover) in a 70:30 forage-to-concentrate ratio. The treatments were a control group receiving the basal diet without additives; a monensin-added diet containing 40 mg/kg of dry matter (DM); a yeast-added diet with *Saccharomyces cerevisiae* at 2 × 10^8^ CFU/g of DM; a NB clay-added diet at 5 g/kg of DM; and MNB diets added at two levels (0.5 g/kg of DM (MNB_Low_) and 1 g/kg of DM (MNB_High_)), with or without *S. cerevisiae*. MNB showed a smaller particle size and improved properties, such as higher conductivity, surface area, and cation exchange capacity, than NB. Sulfur and related functional groups were detected only in MNB. No differences were observed in total GP, while both the monensin diet and the MNB_High_-with-yeast diet significantly reduced CH_4_ emissions compared to the control (*p* < 0.05). The MNB_High_-without-yeast combination significantly (*p* < 0.05) reduced hemicellulose degradation, as well as total protozoal counts, including *Isotricha* and *Epidinium* spp. (*p* < 0.05), compared to the control. Ammonia levels did not differ significantly among treatments, while NB and MNB_High_ diets tended to have (*p* = 0.063) the highest short-chain fatty acid (SCFA) concentrations. These findings suggest the potential modulatory effects of yeast and MNB on rumen fermentation dynamics and CH_4_ mitigation.

## Simple Summary

Reducing greenhouse gas (GHG) emissions from farm animals is important for protecting the environment. In this study, we developed a special form of natural clay, called modified nano-bentonite (MNB), and combined it with a type of yeast (*Saccharomyces cerevisiae*) commonly used in animal diets. We tested whether this mixture could reduce ruminal methane (CH_4_) production without adverse effects on ruminal nutrient degradability in a high-fiber diet. In this study, MNB was developed by organically modifying natural bentonite with sodium dodecyl sulfate (SDS). Our results showed that the combination of MNB with yeast helps lower CH_4_ emissions and improves nutrient degradability, which could be a natural and eco-friendly alternative to traditional antibiotics used in animal farming.

## 1. Introduction

Greenhouse gas (GHG) emissions play a significant role in accelerating climate change and contributing to global warming [1]. The animal production sector represents the largest proportion of GHGs from agriculture, through the emission of methane (CH_4_) and (CO_2_) from the ruminal fermentations and manure of farm animals [2]. Methane is continually accumulating, and the warming potential of CH_4_ is 28 times more than that of CO_2_ [3]. Moreover, CH_4_ emissions from ruminants represent a notable energy loss from the diet, energy that could otherwise be used to enhance meat or milk production [4]. Emissions of GHG can considerably impact climate change and global warming [3]. To mitigate enteric CH_4_ emissions, while improving productivity and animal health, various antimicrobial feed additives, such as ionophores (e.g., monensin), have been commonly used in ruminant nutrition. Monensin, as an ionophore antibiotic, exerts its antimicrobial effect primarily by disrupting bacterial cell membrane integrity and interfering with ion transport, particularly through the exchange of protons for sodium and potassium ions [5]. It selectively alters the ruminal microbial population, including a reduction in certain protozoal groups, and may increase the relative digestion of N postruminally [6]. Furthermore, monensin has been shown to shift starch and nitrogen digestion from the rumen to the postruminal tract [2,6]. However, in high-fiber diets, the effects of monensin on fiber digestibility and nutrient use are not consistent across studies and appear to be influenced by several factors, including the type of forage, dietary composition, and monensin inclusion level [1,2,4,5]. Methane production in the rumen is closely linked to the extent of organic matter (OM) degradation, as higher OM degradation provides more hydrogen for methanogenesis [1,2]. Monensin may reduce CH_4_ emissions by limiting OM fermentation, particularly via its effect on fiber digestion. Nevertheless, this strategy may compromise overall ruminal digestibility [4,5]. Consequently, the search for feed additives that can effectively reduce CH_4_ emissions without impairing OM degradation remains a key challenge in ruminant nutrition. For instance, probiotics, such as *Saccharomyces cerevisiae*, have been shown to enhance fiber degradation by stimulating fibrolytic microbial activity, yet findings regarding their effectiveness in mitigating ruminal CH_4_ emissions remain inconclusive [6].

Clays use is generally sanctioned as safe for both humans and animals [7,8]. Natural bentonite (NB) is often preferred over other types of clay due to its wide availability, cost-effectiveness, high surface area, fine particle size, and strong ion exchange capacity. [8,9] Unlike ionophores, bentonite does not inhibit fiber-degrading microbes; instead, it may improve ruminal fermentation by binding toxins, stabilizing rumen pH, and reducing ammonia [10]. Therefore, NB is extensively used as a feed additive for ruminant nutrition. However, natural bentonite has lower anti-methanogenic effects than other clays [8,9]. Recently, the modification of smectite clays (e.g., bentonite) using cationic and anionic surfactants was shown to enhance their ion exchange capacity and exhibit greater anti-methanogenic activity compared to unmodified clays and ionophores [4,11]. These results are primarily attributed to the enhanced physicochemical properties of the modified clay, particularly the frequency shifts observed in its active functional groups [11]. The modification of smectite clays with organosulfur compounds, such as SDS, alters the smectite clays’ surface properties from hydrophilic to hydrophobic, increases their adsorption potential, and increases their ion exchange capacity. In our previous study, SDS-modified montmorillonite (the major component of bentonite) at the nano scale significantly reduced CH_4_ production by approximately 38% when included at 0.5 g/kg of dry matter in a 50:50% concentrate–forage diet without adverse effects on nutrient degradability [4]. It seems that these modified clays containing sulfur or sulfate are a novel potential approach to alternate ionophores in reducing CH_4_ emission from ruminants; however, their impact on ruminal fermentation and CH_4_ mitigation in high-fiber diets remains unexplored [12,13,14].

The combination of *Saccharomyces cerevisiae* with modified nano-bentonite clay is hypothesized to synergistically enhance ruminal fermentation and mitigate CH_4_ production in high-fiber diets. While *S. cerevisiae* promotes rumen stability by enhancing fiber degradability and elevating ruminal pH [6,15], the modified nano-bentonite (MNB), with its enhanced ion exchange capacity and adsorptive properties, may complement yeast activity by directly inhibiting methanogens and binding fermentation by-products that favor CH_4_ formation [11,13,14]. Therefore, the combined addition is expected to improve fiber degradation, optimize the rumen microbial balance, and achieve greater CH_4_ mitigation than either additive alone. This study aims to develop and evaluate both the physicochemical characteristics and the nutritional impacts of MNB, added with or without *Saccharomyces cerevisiae*, in comparison to natural bentonite (NB) and the ionophore monensin on ruminal fiber degradability and CH_4_ production.

## 2. Materials and Methods

The experimental work was carried out at the Nanotechnology and Greenhouse Gases Laboratory, Department of Animal and Fish Production, Faculty of Agriculture, Alexandria University.

### 2.1. Experimental Feed Additives

Natural bentonite (NB; 95% purity; Egypt Bentonite and Derivatives Co., Alex., Egypt) was organically modified with SDS (Sigma-Aldrich Co., Irvine, Scotland) according to the method described by Soltan et al. [11]. Milling was performed using a photon ball mill (Model PH-BML 912; Photon Scientific Co., Qalyub, Egypt) operating at a revolution speed of 300 ± 10% rpm and a jar rotation speed of 450 ± 10% rpm. The process was carried out for 60 min using a 100 mL zirconia jar filled with zirconia milling balls. An identified yeast inoculum of exogenous *Saccharomyces cerevisiae* was obtained commercially (2 × 10^10^ CFU/g; Allgau Vet. Ltd. Co., Kempten, Germany), while monensin sodium was obtained from Rumensin, Elanco, Itapira, São Paulo, Brazil (with 100 mg/kg purity).

### 2.2. Physicochemical Parameters of NB and MNB Clays

Cation exchange capacity (CEC) was determined following the protocol described by Rhoades [16], using 1 M sodium acetate and 0.1 M sodium chloride solutions. The pH and electrical conductivity (EC) of the experimental feed additives were assessed using a suspension of the clay products in distilled water at a 1:2.5 ratio, measured with a multi-parameter pH meter (GLP-21 model; CRISON, Barcelona, Spain) [17]. The particle size distribution and specific surface area (SSA) were analyzed using a BT-9300S laser particle analyzer (S3 Plus; Dandong Bettersize Scientific Ltd., Dandong, China) [14].

A transmission electron microscope (TEM; JSM1400 plus; JEOL, Los Angeles, CA, USA) was used to obtain dimensional images to detect the size and shape of the clays’ particles. The functional groups of the experimental clays were identified by Fourier transform infrared spectroscopy (FTIR) with an infrared spectrometer (Shimadzu-FTIR-8400S; Osaka, Japan) equipped with a deuterated triglycine sulfate (DTGS) KBr detector and a purge gas generator [4]. To identify the elemental composition of the experimental clays, samples were subjected to an energy-dispersive X-ray (EDX) system coupled with a scanning electron microscope (SEM; Jeol JSM-6360 LA, Jeol Ltd., Akishima, Tokyo, Japan). The SEM analysis was also used to determine the nanoparticle shape of the experimental clay products after coating with gold to improve the imaging of the samples, as described by Soltan et al. [11].

### 2.3. Substrate and Treatments

The substrate used in the in vitro assay consisted of 700 g/kg of DM concentrate and 300 g/kg of DM berseem clover hay (*Trifolium alexandrinum*, fourth cutting). This feed substrate was formulated to meet the nutritional requirements of growing cattle [18]. The chemical composition of the basal diet was determined [19] for DM and ash content, and organic matter (OM) was calculated as the difference between DM and ash, crude protein (CP), and ether extract (EE). Fiber fractions, including neutral detergent fiber (NDF), acid detergent fiber (ADF), and lignin, were analyzed according to the protocol of Van Soest et al. [20]. A semi-automated fiber analyzer (FA-12 fiber analyzer unit; Smart Lab, Cairo, Egypt) was used to determine plant cell wall components sequentially, using the same sample enclosed in F57 filter bags (ANKOM-Technology, Macedon, NY, USA). The composition and chemical characteristics of the basal diet are detailed in Table 1.

The treatments consisted of the addition of test additives as follows: substrate without additives (control); a monensin-added diet containing 40 mg of monensin per kg of DM (monensin); a yeast-added diet with *Saccharomyces cerevisiae* at 2 × 10^8^ CFU/g of DM; a NB clay-added diet at 5 g/kg of DM; and MNB diets added at two levels (0.5 g/kg of DM (MNB_Low_) and 1 g/kg of DM (MNB_High_)), with or without *S. cerevisiae* (2 × 10^8^ CFU/g DM). The doses of monensin and *S. cerevisiae* were selected based on the manufacturers’ recommendations, while the NB dose was adopted from Maki et al. [10], and the MNB inclusion levels were determined according to Soltan et al. [4].

### 2.4. Gas Production (GP) Protocol

#### 2.4.1. Ruminal Inocula: Collection, Handling, and Preparation

In vitro GP was performed in one day using a semi-automatic system [21]. Rumen fluid used for the in vitro assay was obtained separately from three crossbred cow calves (455 ± 10 SE kg) that had been fasted prior to slaughter at the slaughterhouse of the Faculty of Agriculture, Alexandria University, Egypt. The collected ruminal contents were transferred into pre-warmed (40 °C) thermo-containers under continuous flushing with CO_2_ to maintain anaerobic conditions. The contents were then homogenized individually by blending for 10 s, followed by filtration through three layers of cheesecloth. The resulting ruminal inocula were maintained in a water bath at 39 °C with continuous CO_2_ flushing to sustain microbial viability.

For each treatment, 12 in vitro 120 mL incubation bottles (4 bottles/ inoculum) were prepared. A 500 mg sample of each diet was placed into ANKOM fiber bags (ANKOM Technology, Macedon, NY, USA), which were then inserted into incubation bottles containing 15 mL of the prepared rumen inoculum and 30 mL of Menke’s buffer solution, achieving a total headspace volume of 75 mL. For each inoculum, blank flasks containing Menke’s buffered medium and rumen fluid were used to determine the net GP, while standard flasks, containing the same medium, rumen fluid, and clover hay, were included to adjust for sensitivity differences among inocula [21]. The bottles were then sealed with 20 mm butyl rubber stoppers and aluminum crimps to ensure airtight conditions. Incubation was carried out for 48 h at 39 °C to simulate ruminal fermentation.

#### 2.4.2. Gas Determination

The gas pressure within the incubation bottles was recorded using a pressure transducer and a data logger (Pressure Logger-PSI-V2; Smart Lab, Cairo, Egypt) [4]. To determine CH_4_ production, 5 mL of headspace gas was sampled from each bottle at each gas pressure measurement time using a 3 mL syringe. The collected gas samples were then stored in 5 mL vacutainer tubes (Vacutainer^®^ Tubes, Dickinson and Company, Franklin Lakes, NJ, USA) for subsequent analysis. The CH_4_ concentrations were assessed using a laser Gas-pro detector (Gas-Pro-IR-W368334/01-001; Crowcon Detection Instruments Ltd., Abingdon, UK) at each measuring time. Before gases measurements, the detector was calibrated using a certified standard gas mixture following the ISO 9001 quality assurance guidelines and the procedures outlined in BS EN ISO 6145-1:2008 [22].

#### 2.4.3. Nutrient Degradability

Nutrient degradability was determined according to Blümmel et al. [23]. The incubation bags were taken out and immediately placed on ice to stop microbial fermentation, treated with a neutral detergent solution, maintained at 90 °C for 1 h in fiber smart analyzer (FA-12 fiber analyzer unit; SmartLab, Cairo, Egypt), and then washed, dried (70 °C for 48 h), and weighed. The bags were then treated with an acid detergent solution for 1 h, followed by rinsing, drying, and weighing. To determine the acid detergent lignin (ADL) content, all bags were subjected to 72% sulfuric acid treatment, and subsequently, the dried residues were ashed using pre-weighed, ash-free crucibles at 600 °C for 2 h to remove organic components.

The truly degraded OM (TDOM) was calculated by subtracting the amount of undegraded OM from the total incubated amount [23]. Similarly, the degraded portions of NDF and ADF were estimated by the difference between the incubated and the remaining undegraded fiber contents. Cellulose content was determined by subtracting ADL from NDF, while hemicellulose was calculated as the difference between NDF and ADF.

#### 2.4.4. Ruminal Fermentation and Protozoal Count

The final pH values of ruminal samples were measured within 2–3 min of sample collection using a pH meter (GLP-21; CRISON, Barcelona, Spain). An equal volume (2 mL) of rumen fluid and methyl green–formalin–saline solution was combined and stored in a glass container for microscopic analysis of protozoal numbers and identification. The differentiation of the protozoal populations was carried out using a Digital Zoom Video microscope (LCD-3D; GiPPON, Tokyo, Japan) and a brightline hemacytometer counting chamber (Paul-Marienfeld GmbH and Co. K.G., Baden-Württemberg, Germany) [24]. Ammonia concentrations were determined calorimetrically using an enzymatic commercial kit (Biodiagnostic Inc., Giza, Egypt) at a wavelength of 635 nm using a spectrophotometer (E-2100V; PEAK-Instruments, Houston, TX, USA). SCFAs were quantified according to De Baere et al. [25] using high-performance liquid chromatography (HPLC) (Agilent Technologies, Inc., Santa Clara, CA, USA). Mobile phase A was composed of 10 mM KH_2_PO_4_, adjusted to pH 2.4 using phosphoric acid, while acetonitrile was used as mobile phase B. The SCFA separation conditions have been detailed by Soltan et al. [4].

### 2.5. Statistical Analysis

The in vitro assay was conducted in a one-day assay that included all experimental treatments, using three different rumen inocula as true statistical replicates. For each inoculum, four fermentation bottles were incubated per treatment as analytical replicates. These analytical replicates were averaged to provide a single value per treatment per inoculum. Thus, the statistical unit was the mean of the four analytical replicates for each of the three inocula (*n* = 3), representing the true experimental replicates used for statistical analysis. Data were subjected to one-way ANOVA using a general linear model with diet as a fixed effect and rumen inoculum as a random effect. The MIXED procedure in SAS (version 9.0; SAS Institute Inc., Cary, NC, USA) [26] was used with the following statistical model: Yij = μ + Di + Rj + eij, where Yij is the observed response, μ is the overall mean, Di is the fixed effect of the diet, Rj is the random effect of the ruminal inoculum, and eij is the residual error. Treatment means were separated by Tukey’s test, with differences deemed significant at *p* ≤ 0.05 and trends noted at *p* < 0.10.

## 3. Results

### 3.1. Physicochemical Properties of Bentonite Clays

Table 2 presents a comparative analysis of the physicochemical properties of NB and MNB. The results revealed significant alterations in key parameters following modification. MNB clay exhibited a notable reduction in particle size distribution in the nano particle size, with D10, D50, and D90 values substantially lower than those of NB. The pH of MNB had higher alkalinity values (15.51%) compared to that of NB. Similarly, MNB had numerical increases in the values of EC, CEC, and SSA compared to NB.

**Figure 1:**
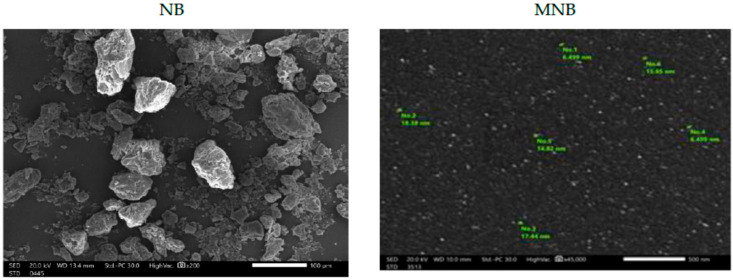
Surface morphology analysis of natural bentonite (NB) and modified nano-bentonite (MNB) using scanning electron microscopy (SEM).

Figure 1 illustrates the surface morphology differences between NB and MNB by SEM analysis. NB showed aggregated particles with irregular shapes and a rough, non-uniform surface. In contrast, modified MNB exhibited well-dispersed, individually separated nano-particles that were smaller, were more uniformly distributed, and presented a smoother surface morphology.

**Figure 2:**
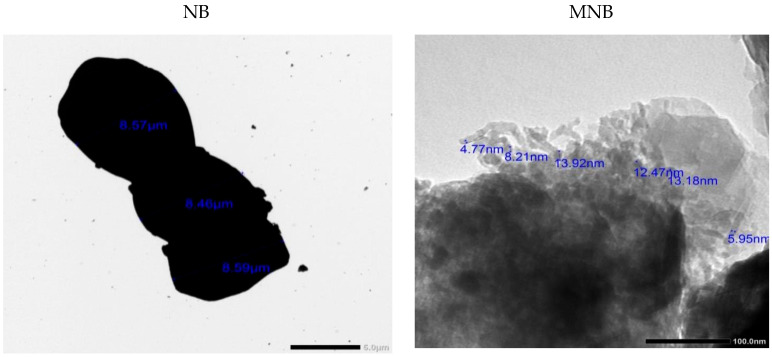
Transmission electron microscope images of natural bentonite (NB) and modified nano-bentonite (MNB).

Figure 2 shows the TEM images of NB and MNB. The TEM micrographs revealed the internal structure and nanoscale features of NB and MNB. MNB particles exhibited a well-defined, nanoscale morphology, with particle sizes ranging from 4.77 to 13.92 nm. In contrast, TEM analysis indicated NB in a highly agglomerated form, with larger, irregular clusters compared to MNB.

Table 3 provides the elemental compositions of NB and MNB using energy-dispersive X-ray spectra. Notably, the presence of sulfur (S) was detected only in MNB, while NB showed no detectable S content; MNB exhibited a concentration of 0.15 ± 0.03 atomic % of S. In contrast, Cl was detected only in NB and was absent in MNB.

Table 4 presents the FTIR results for both NB and MNB, highlighting significant peak maxima (cm^−1^) for various functional groups. In NB, hydroxyl groups (-OH) appeared in the peaks at 3417.46 cm^−1^ and 1633.79 cm^−1^. A Si-O stretching vibration peak appeared at 1031.65 cm^−1^, and an Al-O stretching vibration peak appeared at 913.62 cm^−1^. Peaks 5, 6, 7, and 8 at 796.52 cm^−1^, 695.40 cm^−1^, 538.58 cm^−1^, and 469.73 cm^−1^, respectively, represent additional bending and stretching vibrations characteristic of the bentonite mineral structure. In contrast, MNB exhibited noticeable shifts and alterations in peak positions compared to NB. Notably, peak 2 shifted to 3626.14 cm^−1^. Peak 3 shifted to 1650.96 cm^−1^. Peak 4 shifted to 1186.27 cm^−1^. Of particular significance was the emergence of new peaks in MNB, not observed in NB. For instance, of sulfur-containing functional groups appeared at peak 5 at 639.06 cm^−1^ and peak 6 at 507.93, which was not observed in NB.

### 3.2. Total Gas and CH_4_ Production

Table 5 presents the effects of monensin, NB, *Saccharomyces cerevisiae*, and MNB with or without yeast on the ruminal GP, CH_4_ production, and nutrient degradability. The total GP values ranged from 99.98 to 114.1 mL/g of DM among the treatments, with no significant differences observed (*p* = 0.1228). CH_4_ production (mL/g of TDOM) and truly degraded hemicellulose (TDH) were significantly lower (*p* < 0.05) in monensin and MNB_High_–yeast treatments than in the control. The lowest CH_4_ (in relation to TDH) levels were observed in the MNB_High_-with-yeast group.

The supplementation with MNB, particularly in combination with yeast, significantly enhanced (*p* < 0.05) nutrient degradability compared to the control and other treatments. The highest values of TDOM, TDNDF, TDADF, cellulose, and hemicellulose were observed with MNB supplementation.

### 3.3. Ruminal Fermentation Parameters and Protozoal Count

Results in Table 6 show that ruminal pH remained within the optimal range (6.3–6.4) across treatments, and the MNB_High_-without-yeast combination tended to have the highest ruminal pH values (*p* = 0.0694). Ammonia concentrations were not significantly different among treatments. Protozoal populations exhibited notable shifts, particularly with MNB_High_ supplementation. Total protozoa counts were significantly reduced (*p* = 0.0001) by both MNB levels combined with yeast compared to the control. Also, both levels of MNB combined with yeast reduced (*p* = 0.0004) the *Isotricha* count compared to monensin. All feed supplements (NB, yeast, and MNB) decreased (*p* = 0.0181) the *Epidinium* population compared to monensin, except for the MNB_high_ dose without yeast, which did not show a reduction. Total SCFAs production tended to be higher (*p* = 0.063) by the NB and MNB_High_-without-yeast combination diets than the monensin diet. The individual molar proportions of SCFAs remained stable across treatments.

## 4. Discussion

The results of this study illustrate a comparative analysis of physicochemical properties between NB and MNB. They reveals significant alterations in key parameters following modification. The modification of MNB using SDS led to notable structural changes, including a narrower particle size distribution, reduced particle dimensions, and an enhanced SSA relative to NB clay. The increased surface area is primarily due to the finer particle size achieved through milling, which offers a high surface-to-volume ratio—a characteristic known to enhance reactivity and biological activity, including antimicrobial effects [14]. Additionally, the modified clay exhibited a higher CEC than the natural form, which reflects a greater capacity for ion exchange and the potential for improved adsorption of metal ions within the clay matrix, thereby enhancing the functional performance of MNB. Also, MNB clay exhibited a higher EC than NB. This may be due to several key factors; the reduction in particle size to the nanoscale significantly enhances the surface area and reactivity of MNB, providing more active sites for ion exchange [14,27]. Structural changes during modification by SDS, such as interlayer expansion, also allow for greater mobility and release of exchangeable cations. In addition, MNB showed improved dispersion in aqueous media compared to NB, which facilitates more uniform ion distribution and mobility, collectively resulting in a higher EC [14]. A high EC is often related to a high CEC, as the CEC reflects clay’s ability to absorb and exchange cations, depending on the number of negatively charged sites in its structure. In contrast, the EC measures the actual levels of free ions in solution that can conduct electricity. Clays with a high CEC, such as MNB, typically release more exchangeable cations into the surrounding medium, thereby increasing the EC [4,11,14].

The modification of bentonite clay using SDS was confirmed through SEM and EDX analyses. The SEM analysis showed that the morphological shapes of NB and MNB are markedly different, as conventional NB showed larger, irregular particles with rough and uneven surfaces, whereas MNB exhibited more uniform particles with a smoother texture. The nanoscale structure of MNB was associated with a significantly higher SSA, as demonstrated by the results. The improved homogeneity and smaller particle size observed in MNB likely result from the modification method, which is known for producing well-dispersed and consistent nanoparticles [4,7,12]. Particle size distribution analysis supported the SEM observations, showing a much narrower size range for MNB compared to NB. These refined surface features can enhance the functional properties of MNB, such as adsorption capacity and catalytic activity, due to increased surface reactivity [14].

Similarly, TEM analysis confirmed the information regarding the size and structural features of both NB and MNB particles. MNB displayed a clearly defined nanoscale structure, with particle diameters measured from 4.77 to 13.92 nm. In NB, the TEM images exhibited a relatively coarse and irregular particle arrangement typical of natural minerals, with discernible layering indicative of its laminar structure. Conversely, MNB displayed a more refined and uniform morphology, characterized by finer particle sizes and a greater degree of dispersion, suggestive of the successful modification process [28].

The high CEC and EC of the modified clay may confirm the ion exchange process between the unmodified clay and the SDS ionic surfactant. The changes in the mineral composition detected by the EDX analysis supported this suggestion. During the modification process using an ionic surfactant such as SDS, some of the native ions of the clay are partially replaced or displaced by the surfactant’s functional groups [29]. Thus, the appearance of S exclusively in MNB can be attributed to the successful incorporation of SDS into the clay structure. Since SDS contains sulfur in its sulfate group (–SO_4_^2−^), its detection via EDX and FTIR in MNB confirms the presence of the surfactant and supports evidence of intercalation or surface adsorption. This chemical modification alters the elemental profile of the clay, reducing native ions like Ca and Na, while introducing new elements, such as S [11,28]. The presence of S and sulfur-containing functional groups in MNB may enhance the antimicrobial, antifungal, and cytotoxic activities of the modified clay, which could have implications for its application as a feed additive in ruminal fermentation [4,11].

The pH of MNB indicates a shift toward alkalinity compared to the neutral pH of NB. The increased oxygen content (detected by EDX) and the presence of distinct FTIR peaks of OH^−^ groups in MNB may directly contribute to the rise in pH, enhancing MNB’s alkalinity [27,28,29]. Moreover, the high ion exchange of MNB may promote a more basic environment, as monovalent ions like Na^+^ are more mobile and readily interact with water molecules to increase OH^−^ [3]. Furthermore, the structural rearrangement and increased interlayer spacing in MNB facilitate stronger interactions with water, encouraging further dissociation and the release of hydroxyl ions [29]. Collectively, these chemical and structural changes enhance the alkaline properties of MNB compared to NB.

The results of the in vitro GP experiments indicated no significant differences among the effects of the experimental diets on ruminal GP, which is inconsistent with our previous findings in which modified clays could reduce the total gas production in 50:50 concentrate-to-forage diets [4,6,11]. Notably, in these studies, the values of the total GP in the 50:50 forage-to-concentrate diets were higher than what were obtained in our study, supporting the notion that the effects of modified clays are influenced by diet type, fermentation dynamics, and substrate availability. In the 50:50 diets, the higher concentrate level provides more readily fermentable carbohydrates, leading to faster microbial fermentation and greater GP under normal conditions. In contrast, the 70:30 forage diet in this study primarily relies on fiber fermentation, which is inherently slower and produces less gas [20,30].

Similar CH_4_ reductions (ml/g of TDOM) were observed in monensin and MNB_High_–yeast diets. However, the reduction caused by the monensin diet was accompanied by decreased degradability of OM and cellulose. Notably, the use of MNB clay, whether combined with yeast or not, resulted in improved degradability of OM and fiber fractions. Hydrogen ions (H^+^) are key intermediates generated during the microbial degradation of OM and fiber, and they serve as essential substrates for methanogens to convert CO_2_ into CH_4_ [2,3]. Thus, our findings suggest that the observed reduction in CH_4_ production was most pronounced with MNB_High_ with yeast. These results indicate that the CH_4_-lowering effect was not due to a general suppression of overall ruminal microbial activity but may, instead, be specifically linked to modifications in the methanogenesis pathway. This hypothesis was also observed in our previous work using a 50:50 concentrate-to-forage diet [4,11]. The notable shifts in absorption bands related to OH and sulfate groups, along with an increased CEC in MNB compared to NB clay, suggested enhanced H^+^ binding potential. Additionally, the presence of S–S bonds in MNB could contribute to anti-methanogenic effects, as sulfur- and sulfate-based compounds can compete with methanogens for H^+^, promoting the formation of hydrogen sulfide (H_2_S) rather than CH_4_ [31,32].

Nutrient degradability showed that supplementation with MNB, particularly in combination with yeast, significantly enhanced nutrient degradability compared to the control and other treatments. The highest values of TDOM, TDNDF, TDADF, cellulose, and hemicellulose were observed with MNB supplementation, indicating improved fiber degradation efficiency. The superior degradability observed with MNB can be attributed to its physicochemical properties. The increased surface area, porosity, and cation exchange capacity of MNB likely enhanced its adsorption and buffering capacity, creating a more favorable ruminal environment for microbial activity. Additionally, its ability to bind fermentation inhibitors, while providing essential minerals, may have contributed to the improved digestion of structural carbohydrates [4]. *S cerevisiae* also can stimulate the growth of other H_2_-consuming microbes, thereby diverting H away from methanogenesis [11,29]. Moreover, yeast enhances rumen conditions (e.g., stabilizing pH, scavenging oxygen), creating a favorable environment for cellulolytic bacteria [6,12].

Results of ruminal fermentation show that ruminal pH remained within the optimal range (6.3–6.4) across treatments, with minor variations. The slightly higher pH observed in the MNB_High_ diet suggests enhanced fermentation activity, potentially linked to improved microbial efficiency and, consequently, nutrient degradability. The reductions in ruminal pH with MNB_Low_ with yeast may likely result from the synergistic enhancement of microbial fermentation and acid production, without sufficient buffering capacity at the low MNB level to offset the increased acid load.

Ammonia concentrations were not significantly different among treatments, indicating that all the experimental feed additives did not adversely affect protein degradation or nitrogen metabolism [12]. Protozoal populations exhibited notable shifts, particularly with MNB_High_ supplementation. Total protozoa were significantly reduced in the MNB-high groups, with pronounced decreases in *Isotricha* and *Epidinium* species. It is worth noting that the protozoal population remained high in the yeast-only diet, but its reduction was observed when yeast was combined with modified MNB. *Saccharomyces cerevisiae* is known to provide growth factors as vitamins (especially B-complex) and amino acids, which can support the proliferation of most rumen protozoa [6,30]. Yeast also scavenges oxygen, creating a more favorable anaerobic environment that benefits protozoa [30]; thus, it remained high in the yeast diet. This reduction is likely due to the high CEC and adsorption properties of MNB, which may have affected protozoal attachment sites or indirectly altered their ecological balance by modulating hydrogen availability [3]. Also, it can be suggested that MNB may adsorb bioactive compounds released by yeast, reducing their availability and thus blunting yeast’s positive effects on protozoal proliferation [12]. The reduced protozoal numbers align with lower CH_4_ emissions, reinforcing the known link between protozoa and methanogenesis.

The total SCFA production was higher in the NB and MNB_High_ treatments than in the monensin diet, suggesting enhanced fermentation activity [3]. The improved SCFA concentrations in MNB-supplemented groups can be attributed to the high SSA and CEC of nano-bentonite, which likely facilitated microbial attachment and metabolic efficiency [29,30]. This increase aligns with the enhancing of the fiber fraction degradability caused by MNB treatments [1]. Unexpectedly, neither acetate nor propionate proportions changed among the treatments, as fatty acids are linked to enhanced fibrolytic and amylolytic microbial activity. These results are inconsistent with our previous studies, where clays modified with SDS shifted the fermentation pathway toward increased propionate production using a 50:50 forage-to-concentrate diet [4,11,12]; nonetheless, this effect did not occur in our findings. It seems that the effects of the modified clays on SCFA production appear to be mainly influenced by the diet type (i.e., fiber content).

## 5. Conclusions

The results demonstrated that MNB possesses superior physicochemical characteristics compared to NB. MNB, particularly when combined with *Saccharomyces cerevisiae* yeast, can be a promising alternative to monensin for improving rumen fermentation in high-forage diets. MNB improved OM degradation at both inclusion levels, and CH_4_ mitigation was more evident at higher inclusion levels, especially when combined with yeast. However, the effects on protozoal populations, including *Epidinium* and *Isotricha*, differed between monensin and MNB treatments, with MNB showing distinct interaction patterns with yeast that warrant further investigation. While MNB_High_ without yeast increased total SCFA concentrations compared to monensin, none of the additives altered the molar proportions of individual SCFAs. Given these findings, MNB, especially in combination with yeast, may serve as an effective CH_4_ mitigation strategy. However, further in vivo studies are needed to validate these in vitro observations, assess long-term impacts on animal health and productivity, and better understand the microbial dynamics underlying these effects.

## Figures and Tables

**Table 1:** Ingredients and chemical composition (expressed on a DM basis) of the basal substrate used in the in vitro assay.

	Experimental Substrate Diet
Item	(g/kg DM)
Ingredients	
Ground maize	70
Wheat bran	65
Soybean meal	20
Barley	110
Brocken rice	25
*Trifolium alexandrinum* clover	700
Calcium carbonate	5
Sodium chloride	3
Vitamins and minerals mixture ^1^	2
Chemical composition	
Organic matter	955
Crude protein	14.9
Ether extract	12.00
Neutral detergent fiber	604
Acid detergent fiber	320
Acid detergent lignin	65
*Cellulose*	255
*Hemicelluloses*	284

^1^ Each kilogram of the supplement provided the following: 80 mg of manganese, 60 mg of zinc, 35 mg of iron, 8 mg of copper, 0.6 mg of selenium, 600 mg of choline chloride, 3 mg of vitamin B6, 3 mg of thiamine, 1.0 mg of folic acid, 10 μg of vitamin B12, 12,000 IU of vitamin A, 2500 IU of vitamin D_3_, 20 IU of vitamin E, 50 μg of d-biotin, 1 mg of calcium pantothenate, 50 mg of nicotinic acid, 1.3 mg of menadione, and 5.5 mg of riboflavin.

**Table 2:** Physicochemical characteristics of natural bentonite (NB) and modified nano-bentonite (MNB).

	Bentonite Feed Additives		Difference (%) *
Item	NB	MNB	
Particle size distribution			
D10 (nm)	24,090	42	−99.83
D50 (nm)	37,970	57	−99.85
D90 (nm)	63,230	72	−99.89
pH	7.26	8.383	+15.51
Electrical conductivity (ppm)	125	519	+315.2
Cation exchange capacity (meq/100 g)	77.6	145.65	+87.7
Specific surface area (m^2^/g)	0.411	36.282	+8729.9

* Percentage differences (%) were calculated to indicate the relative change in NB and MNB.

**Table 3:** Determination of the elemental compositions of natural bentonite (NB) and modified nano-bentonite (MNB) using energy-dispersive X-ray spectra.

	Bentonite Feed Additives		Difference (%) *
Item	NB	MNB	
Element (atomic %) *			
O^−2^	51.75 ± 0.49	56.51 ± 0.42	+9.20
Na^+1^	1.85 ± 0.11	1.08 ± 0.08	−41.62
Mg^+2^	1.42 ± 0.09	1.53 ± 0.08	+7.75
Al^+3^	9.71 ± 0.19	9.49 ± 0.16	−2.27
Si^+4^	24.49 ± 0.31	23.46 ± 0.26	−4.20
Cl^−1^	0.88 ± 0.07	0	−100.00
S^+6^	0	0.15 ± 0.03	+0.15
K^+1^	1.07 ± 0.08	0.80 ± 0.06	−25.23
Ca^+2^	0.60 ± 0.07	0.44 ± 0.05	−26.67
Ti^+3^	1.05 ± 0.08	0.79 ± 0.06	−24.76
Fe^+2^	7.19 ± 0.24	5.75 ± 0.18	−20.03

* Percentage differences (%) were calculated to indicate the relative change in NB and MNB.

**Table 4:** Fourier transform infrared spectroscopy (FTIR) of natural bentonite (NB) and modified nano-bentonite (MNB).

	Bentonite Feed Additives (Peak Maxima (cm^−1^))	
Peak No	NB	MNB
1	3417.46	3646.24
2	1633.79	3626.14
3	1031.65	1650.96
4	913.62	1186.27
5	796.52	639.06
6	695.40	507.93
7	538.58	486.61
8	469.73	463.26

**Table 5:** Supplementation effects of monensin, natural bentonite (NB), yeast (*Saccharomyces cerevisiae*), and modified nano-bentonite (MNB) with or without yeast on ruminal gas production (GP), methane (CH_4_) production, and nutrient degradability.

	Treatments (T)									
					Modified Nano-Bentonite					
	Control	Monensin	NB	Yeast	MNB_Low_		MNB_High_		SEM	*p* Value
					With Yeast	Without Yeast	With Yeast	Without Yeast		
GP (mL/g DM)	99.979	108.69	105.6	104.2	114	106.5	114.1	108.16	3.613	0.1228
Methane										
CH_4_ (mL/g TDOM)	8.7712 ^a^	6.2805 ^b^	7.291 ^ab^	7.969 ^ab^	6.869 ^ab^	8.225 ^ab^	6.0727 ^b^	6.953 ^ab^	0.5447	0.0116
CH_4_ (mL/g TDC)	19.57	14.105	18.04	17.89	15.52	17.44	13.790	15.004	1.4773	0.0764
CH_4_ (mL/g TDH)	10.75 ^a^	7.681 ^ab^	8.125 ^ab^	8.78 ^ab^	7.68 ^ab^	9.501 ^ab^	7.289 ^b^	7.68 ^ab^	0.7337	0.0363
Nutrient degradability (g/kg DM)										
TDOM	537 ^b^	555 ^b^	564 ^ab^	557 ^ab^	572 ^a^	572 ^a^	580 ^a^	585 ^a^	7.0176	0.0005
TDNDF	268 ^b^	297 ^ab^	310 ^ab^	299 ^ab^	324 ^a^	323 ^a^	336 ^a^	344 ^a^	11.095	0.0005
TDADF	138 ^b^	152 ^ab^	135 ^b^	154 ^ab^	157 ^ab^	170 ^a^	166 ^ab^	178 ^a^	11.571	0.0644
TDC	239 ^c^	236 ^c^	246 ^b^	251 ^b^	254 ^a^	273 ^a^	258 ^a^	273 ^a^	11.352	0.1924
TDH	414 ^b^	461 ^ab^	507 ^a^	464 ^ab^	512 ^a^	495 ^a^	531 ^a^	527 ^a^	16.884	0.0001

SEM = standard error of the mean; DM = dry matter; OM = organic matter; TDOM = truly degraded organic matter; DNDF = degraded natural detergent fiber; DADF = degraded acid detergent fiber; TDC = degraded cellulose; TDH = degraded hemicellulose. ^a,b,c^ Means within a row without a common superscript letter differ significantly at *p* < 0.05.

**Table 6:** Supplementation effects of monensin, natural bentonite (NB), yeast (*Saccharomyces cerevisiae*), and modified nano-bentonite (MNB) with or without yeast on ruminal fermentation parameters and protozoal counts.

	Treatments (T)									
					Modified Nano-Bentonite					
	Control	Monensin	NB	Yeast	MNB_Low_		MNB_High_		SEM	*p* Value
					With Yeast	Without Yeast	With Yeast	Without Yeast		
Ruminal pH	6.378 ^ab^	6.366 ^ab^	6.368 ^ab^	6.353 ^ab^	6.325 ^b^	6.373 ^ab^	6.365 ^ab^	6.386 ^a^	0.013	0.0694
Ammonia (mg/100 mL)	26.69	20.8	26.256	25.35	28.27	26.09	23.69	25.95	2.103	0.3627
Protozoa (10^5^/mL)										
*Entodinium*	3.200	5.200	3.600	5.000	3.400	4.200	3.000	2.800	0.864	0.3818
*Eudiplodinium*	0.8000	1.4000	1.0000	0.600	0.400	0.800	0.600	0.4000	0.3949	0.6602
*Epidinium*	3.600 ^b^	5.400 ^a^	4.600 ^b^	4.800 ^b^	2.000 ^bc^	2.800 ^bc^	1.400 ^c^	5.000 ^a^	0.8921	0.0181
*Isotrica*	10.00 ^ab^	11.33 ^a^	9.167 ^ab^	11.667 ^a^	5.500 ^b^	6.667 ^ab^	4.867 ^b^	7.000 ^ab^	1.1727	0.0004
*Ophryscolex*	0.8000	0.6000	1.0000	0.6000	0.6000	0.0001	0.6000	0.2000	0.3000	0.3752
Total	13.600 ^a^	12.00 ^ab^	11.00 ^ab^	14.000 ^a^	6.600 ^bc^	8.000 ^abc^	4.400 ^c^	8.400 ^abc^	1.4275	0.0001
SCFAs										
Total (mM)	88.64 ^ab^	83.62 ^b^	96.98 ^a^	94.59 ^ab^	93.65 ^ab^	86.71 ^ab^	92.82 ^ab^	97.50 ^a^	2.923	0.063
Acetate (%)	51.246	51.77	51.375	54.988	51.507	52.498	53.130	53.61	1.208	0.3617
Propionate (%)	23.634	26.712	25.07	24.685	26.74	25.811	26.79	26.227	1.147	0.4611
Butyrate (%)	15.48	13.947	13.39	15.16	15.28	14.75	13.814	14.27	0.856	0.5949
Isobutyric (%)	1.7783	2.708	1.602	3.132	1.776	1.6429	1.6669	1.7107	0.656	0.6098
Isovalric (%)	1.423	1.2553	1.8889	0.985	0.8242	1.5815	1.2894	1.4632	0.439	0.7626
Valric (%)	4.6939	5.6265	3.0569	2.627	3.8673	3.7128	3.3069	2.7107	0.701	0.0960

SEM = standard error of the mean; SCFAs = short-chain fatty acids. ^a,b,c^ Means within a row without a common superscript letter differ significantly at *p* < 0.05.

## Data Availability

The original contributions presented in this study are included in the article. Further inquiries can be directed to the corresponding author(s).

## Abbreviations

The following abbreviations are used in this manuscript:

MNB	modified nano-bentonite
NB	natural bentonite
SEM	scanning electron microscopy
TEM	transmission electron microscope
FTIR	fourier transform infrared spectroscopy
GP	gas production
TDOM	truly degraded organic matter
DNDF	degraded natural detergent fiber
DADF	degraded acid detergent fiber
TDC	degraded cellulose
TDH	degraded hemicellulose
